# Effect of arteriovenous access closure and timing on kidney function in kidney transplant recipients

**DOI:** 10.1371/journal.pone.0226309

**Published:** 2019-12-11

**Authors:** Seonjeong Jeong, Hyunwook Kwon, Jee Yeon Kim, Young Hoon Kim, Tae-Won Kwon, Jung Bok Lee, Yong-Pil Cho, Duck Jong Han

**Affiliations:** 1 Department of Surgery, University of Ulsan College of Medicine, Asan Medical Center, Seoul, Republic of Korea; 2 Department of Clinical Epidemiology and Biostatistics, University of Ulsan College of Medicine, Asan Medical Center, Seoul, Republic of Korea; University of Glasgow, UNITED KINGDOM

## Abstract

This study aimed to determine whether the closure of a functioning arteriovenous (AV) access affects the estimated glomerular filtration rate (eGFR) and to compare outcomes according to the timing of AV access closure after kidney transplantation (KT). From 2009 to 2015, medical records were retrospectively reviewed for 142 kidney transplant recipients (KTRs) who underwent AV access closure. The 142 KTRs were categorized into three groups: AV access closure was performed within 6 months after KT in Group 1 (n = 45), at 6–12 months after KT in Group 2 (n = 49), and at 12–24 months after KT in Group 3 (n = 48). The baseline (at the time of AV access closure) and follow-up eGFR values during the 3-year follow-up period were compared. Linear mixed model analysis revealed no significant association between longitudinally observed eGFR values and the amount of time elapsed after AV access closure in the study population (P = 0.36). There was no significant association between 3-year eGFR values and the timing of AV access closure (P = 0.58). In conclusion, after successful KT, AV access closure did not affect the eGFR significantly, and the timing of AV access closure was not significantly associated with outcomes.

## Introduction

Controversy exists regarding the decision of whether to close or preserve a functioning arteriovenous (AV) access for successful kidney transplant recipients (KTRs) [1–6]. Surgical closure is usually performed for patients with specific conditions, including high-flow fistulas, a high-risk cardiovascular status, or cosmetic reasons [3]. Although a functioning AV access for hemodialysis burdens the cardiovascular system with increased cardiac output and pulmonary artery pressure, increasing cardiovascular risk [1], many patients need to return to dialysis, and the physiologic effect of AV access on these patients does not strongly favor routine closure following kidney transplantation (KT) with stable kidney allograft function [2, 3]. Moreover, the effects of a functioning AV access on the estimated glomerular filtration rate (eGFR) and kidney allograft survival are unclear. Concerning the evolution of kidney allograft function, Vajdič et al. [5] showed that the persistence of a functioning arteriovenous fistula (AVF) at 1 year after KT was associated with a lower eGFR and an increased risk of allograft loss. On the other hand, Weekers et al. [3] observed a significant acceleration of eGFR decline over 12 months following the closure of a functioning AVF in KTRs. Furthermore, there is no accepted policy for the timing of AV closure after successful KT.

This study aimed to determine if the closure of a functioning AV access would affect the eGFR and to compare outcomes according to the timing of AV access closure after KT.

## Materials and methods

### Study design and population

This single-center, observational study was conducted retrospectively using data extracted from a prospective KT registry. The study protocol was approved by the institutional review board of Asan Medical Center, Republic of Korea (2019–0177), which waived the need for informed consent because of the retrospective nature of the study.

Between 1 January 2009 and 31 December 2015, 1949 consecutive patients underwent KT for end-stage kidney disease (ESKD) at our hospital: 1511 living-donor KTs (77.5%) and 438 deceased-donor KTs (22.5%). To ensure that we specifically analyzed the effect of the timing of AV access closure on serial changes in the eGFR, we excluded patients who received surgical closure at other hospitals and those who experienced spontaneous AV access thrombosis. We also excluded those with technical failures, those with early graft loss and nonfunctioning kidneys caused by postoperative immunological rejection, and those who died from systemic infection during the 3 years after AV access closure, because these might be potential confounding factors. Among the 155 KTRs who underwent surgical closure of a functioning AV access at our hospital, we also excluded those who were lost to follow-up (n = 3, 1.9%) and 10 foreigners who were followed up in a foreign country (6.5%). Finally, 142 patients (91.6%) were included in the analysis.

### Postoperative immunosuppressive therapy

A detailed description of postoperative conventional immunosuppressive therapy for adult KTRs at our institution has been published previously [7]. During the period under review, the patients were treated with anti-IL-2 receptor antibody (basiliximab) once each on days 0 and 4 for induction. Alternatively, for patients with immunological risk factors, such as highly sensitized individuals or those with previous graft loss due to rejection and those who wished to avoid long-term steroids, an induction regimen of rabbit antithymocyte globulin (Thymoglobulin; Genzyme, Cambridge, MA, USA) was administered. All patients received triple therapy as maintenance immunosuppression at the time of discharge, which consisted of a combination of calcineurin inhibitors (tacrolimus or cyclosporine), a mycophenolic acid derivative, and corticosteroids. Additionally, all patients received trimethoprim-sulfamethoxazole for 6 months as prophylaxis against *Pneumocystis jirovecii*.

### Study outcomes and follow-up

Medical records were systematically reviewed, and the patients were categorized into three groups based on the timing of AV access closure after successful KT: AV access closure within 6 months (Group 1), AV access closure at 6–12 months (Group 2), and AV access closure at 12–24 months (Group 3). At our institution, for patients with stable allograft function, serum creatinine and eGFR values were measured every 6 months after KT. The baseline (at the time of AV access closure) and follow-up (at approximately 6-month intervals) serum creatinine and eGFR values during the 3-year follow-up period were compared regardless of the timing of AV access closure and compared based on the timing of AV access closure (between the three groups). Next, subgroup analysis was performed according to the type of donor graft (living donor and deceased donor). Heart failure (HF) at the time of AV access closure, categorized by systolic or diastolic dysfunction, was diagnosed based on the Framingham score (two major criteria or one major plus two minor criteria) and evidence of systolic or diastolic dysfunction on Doppler echocardiography, as previously described [8–10]. In the BK virus DNA test, the quantification limit was over 100 copies/mL, and the lower limit of linearity was below 1000 copies/mL. BK viremia was diagnosed using a real-time polymerase chain reaction (PCR) assay and the TaqMan probe method. BK virus DNA levels of more than 4 log copies/mL in the blood indicated BK viremia; negative quantitative PCR results indicated the absence of BK virus DNA or a quantitation limit of below 100 copies/mL. The eGFR values were calculated using the Chronic Kidney Disease Epidemiology Collaboration (CKD-EPI) 2009 equation [11].

### Statistical analysis

Categorical variables are reported as frequencies or percentages, and continuous variables are reported as means and standard deviations. To compare baseline and clinical characteristics among the three groups, the chi-square test for categorical variables and ANOVA with Scheffe's multiple comparisons were performed. When there was a significant difference between the three groups, statistical significance was confirmed to identify the differences between all combinations of two groups. Linear mixed model analysis was performed to evaluate the group, time, and interaction between group and time in longitudinally observed eGFR data. A P value of <0.05 was considered statistically significant. Statistical analyses were performed using SPSS version 21.0 (IBM Corp., Armonk, NY, USA).

## Results and discussion

The mean patient age of the study population was 44 years (range, 19–69 years), and 67 patients (47.2%) were men. There were 83 living-donor KTs (58.5%) and 59 deceased-donor KTs (41.5%). The patients were further categorized into three groups based on the timing of AV access closure after KT. Group 1 included 45 patients (31.7%) who underwent AV access closure within 6 months after KT, Group 2 included 49 patients (34.5%) for whom AV access closure was performed at 6–12 months after KT, and Group 3 included 48 patients (33.8%) for whom AV access closure was performed at 12–24 months after KT. **Table 1** shows the baseline and clinical characteristics of the study sample according to the timing of AV access closure. There were no significant differences between the groups in terms of the age, risk factors, type of donor graft (living donor *versus* deceased donor), type of AV access (AVF *versus* arteriovenous graft [AVG]), and baseline serum creatinine and eGFR values, except for the time interval between KT and AV access closure. Among the 142 KTRs, 17.6% (n = 25) required AV access closure for a diagnosis of HF, and there was no significant difference in the proportion of HFs at the time of AV access closure between the groups (P = 0.44). The incidence of BK viremia did not differ significantly between the groups (P = 0.23). In subgroup analysis according to the type of donor graft, there were no significant differences between the groups (among the 83 living-donor KTRs) except for the time interval between KT and AV access closure (**Table 2**). Among the 59 deceased-donor KTRs, there were significant differences in the mean body mass indices between groups 2 and 3 and the time interval between KT and AV access closure between the groups (**Table 3**).

**Table 1:** Baseline and clinical characteristics of the study sample according to the timing of AV access closure.

	Total	Group 1	Group 2	Group 3	P value
Patients (n)	142	45 (31.7)	49 (34.5)	48 (33.8)	
Age (year)	44.0 ± 11.1	44.6 ± 10.1	42.8 ± 12.3	44.5 ± 10.8	0.68
Male	67 (47.2)	23 (51.1)	24 (49.0)	20 (41.7)	0.63
BMI (kg/m^2^)	21.6 ± 2.8	21.5 ± 2.6	21.2 ± 3.0	22.2 ± 2.7	0.23
Diabetes mellitus	28 (19.7)	13 (28.9)	8 (16.3)	7 (14.6)	0.17
Hypertension	108 (76.1)	33 (73.3)	40 (81.6)	35 (72.9)	0.53
CVA	15 (10.6)	6 (13.3)	4 (8.2)	5 (10.4)	0.72
Living donor KT	83 (58.5)	27 (60.0)	27 (55.1)	29 (60.4)	0.84
AVF	134 (94.4)	41 (91.1)	46 (93.9)	47 (92.9)	0.35
HF^a^	25 (17.6)	8 (17.8)	11 (22.4)	6 (12.5)	0.44
BK viremia	11 (7.8)	1 (2.2)	5 (10.2)	5 (10.4)	0.23
Creatinine (mg/dL)^b^	1.2 ± 0.3	1.1 ± 0.2	1.1 ± 0.4	1.2 ± 0.4	0.17
eGFR (mg/min/1.73 m^2^)^b^	71.9 ± 19.1	71.6 ± 18.7	76.4 ± 20.9	67.7 ± 16.8	0.18
KT to AV access closure (month)	10.0 ± 5.6	3.8 ± 1.8	9.3 ± 2.0	16.4 ± 2.7	<0.001^c^
HD to KT (month)	55.7 ± 44.3	50.7 ± 45.0	52.0 ± 41.7	64.2 ± 45.9	0.27

Continuous data are presented as means ± standard deviations; categorical data are presented as numbers (%).

AV, arteriovenous; AVF, arteriovenous fistula; BMI, body mass index; CVA, cerebrovascular accident; eGFR, estimated glomerular filtration rate; HD, hemodialysis; HF, heart failure; KT, kidney transplantation

^a^ HF at the time of AV access closure

^b^ Values at the time of AV access closure

^c^ Significant differences between the three groups

**Table 2:** Baseline and clinical characteristics of the living-donor KTRs according to the timing of AV access closure.

	Total	Group 1	Group 2	Group 3	P value
Patients (n)	83	27 (32.5)	27 (32.5)	29 (34.9)	
Age (year)	44.7 ± 11.1	45.8 ± 10.6	44.2 ± 12.8	44.1 ± 10.1	0.81
Male	46 (55.4)	14 (51.9)	14 (51.9)	18 (39.1)	0.67
BMI (kg/m^2^)	21.8 ± 2.9	21.5 ± 2.5	22.0 ± 3.5	21.9 ± 2.7	0.76
Diabetes mellitus	10 (12.1)	6 (22.2)	1 (3.7)	3 (10.3)	0.11
Hypertension	65 (78.3)	22 (81.5)	23 (85.2)	20 (69.0)	0.33
CVA	12 (14.5)	6 (22.2)	3 (11.1)	3 (10.3)	0.46
AVF	78 (94.0)	25 (92.6)	24 (88.9)	29 (100.0)	0.19
HF^a^	9 (10.8)	4 (14.8)	3 (11.1)	2 (6.9)	0.62
BK viremia	4 (4.8)	1 (3.7)	1 (3.7)	2 (6.9)	>0.99
Creatinine (mg/dL)^b^	1.1 ± 0.3	1.1 ± 0.2	1.1 ± 0.3	1.2 ± 0.4	0.24
eGFR (mg/min/1.73 m^2^)^b^	73.9 ± 17.9	73.6 ± 16.7	77.7 ± 18.2	70.7 ± 18.5	0.15
KT to AV access closure (month)	10.1 ± 5.6	3.8 ± 1.8	9.7 ± 2.0	16.1 ± 2.8	<0.001^c^
HD to KT (month)	37.8 ± 43.4	30.4 ± 35.2	31.1 ± 39.4	50.9 ± 51.5	0.13

Continuous data are presented as means ± standard deviations; categorical data are presented as numbers (%).

AV, arteriovenous; AVF, arteriovenous fistula; BMI, body mass index; CVA, cerebrovascular accident; eGFR, estimated glomerular filtration rate; HD, hemodialysis; HF, heart failure; KT, kidney transplantation

^a^ HF at the time of AV access closure

^b^ Values at the time of AV access closure

^c^ Significant differences between the three groups

**Table 3:** Baseline and clinical characteristics of the deceased-donor KTRs according to the timing of AV access closure.

	Total	Group 1	Group 2	Group 3	P value
Patients (n)	59	18 (30.5)	22 (37.3)	19 (32.2)	
Age (year)	42.9 ± 11.1	42.8 ± 9.3	41.2 ± 11.7	45.1 ± 12.0	0.54
Male	29 (49.2)	8 (44.4)	11 (50.0)	10 (52.6)	0.88
BMI (kg/m^2^)	21.4 ± 2.6	21.6 ± 2.8	20.2 ± 1.9	22.6 ± 2.7	0.012^a^
Diabetes mellitus	18 (30.5)	7 (38.9)	7 (31.8)	4 (21.1)	0.49
Hypertension	43 (72.9)	11 (61.1)	17 (77.3)	15 (79.0)	0.47
CVA	3 (5.1)	0	1 (4.6)	2 (10.5)	0.51
AVF	56 (94.9)	16 (88.9)	22 (100.0)	18 (94.7)	0.19
HF^b^	16 (27.1)	4 (22.2)	8 (36.4)	1 (21.1)	0.47
BK viremia	7 (11.9)	0	4 (18.2)	3 (15.8)	0.17
Creatinine (mg/dL)^c^	1.2 ± 0.3	1.2 ± 0.3	1.2 ± 0.4	1.3 ± 0.3	0.61
eGFR (mg/min/1.73 m^2^)^c^	69.1 ± 20.6	68.4 ± 21.5	74.9 ± 24.1	63.1 ± 13.0	0.72
KT to AV access closure (month)	9.9 ± 5.6	3.9 ± 1.8	8.7 ± 1.8	16.8 ± 2.6	<0.001^d^
HD to KT (month)	80.9 ± 31.7	81.2 ± 41.5	77.7 ± 28.2	84.3 ± 25.8	0.81

Continuous data are presented as means ± standard deviations; categorical data are presented as numbers (%).

AV, arteriovenous; AVF, arteriovenous fistula; BMI, body mass index; CVA, cerebrovascular accident; eGFR, estimated glomerular filtration rate; HD, hemodialysis; HF, heart failure; KT, kidney transplantation

^a^ Significant difference between groups 2 and 3

^b^ HF at the time of AV access closure

^c^ Values at the time of AV access closure

^d^ Significant differences between the three groups

**Fig 1:**
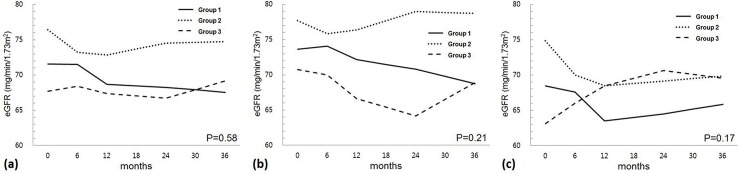
Serial changes in eGFR values according to the timing of AV access closure. **(a)** The entire study sample, **(b)** the living-donor KT group, and **(c)** the deceased-donor KT group. Group 1, AV access closure within 6 months after KT; Group 2, AV access closure at 6–12 months after KT; Group 3, AV access closure at 12–24 months after KT, AV, arteriovenous; eGFR, estimated glomerular filtration rate; KT, kidney transplantation.

In terms of the association between longitudinally observed eGFR values and time after AV access closure, linear mixed model analysis revealed no significant association in either the entire study sample (P = 0.36) or the living-donor (P = 0.37) and deceased-donor (P = 0.90) KT groups (**Table 4**). In the assessment of the association between 3-year eGFR values and the timing of AV access closure, no significant association was observed in either the entire study sample (P = 0.58) or the living-donor (P = 0.21) and deceased-donor (P = 0.17) KT groups (**Fig 1**).

**Table 4:** Correlation between longitudinally observed eGFR values and time after AV access closure in linear mixed model analysis.

	Patients (n)	Baseline eGFR^a^ (mg/min/1.73 m^2^)	3-year eGFR^b^ (mg/min/1.73 m^2^)	P value
Study sample	142 (100.0)	71.9 ± 19.1	70.6 ± 22.2	0.36
Living-donor KT	83 (58.5)	73.9 ± 17.9	72.0 ± 22.7	0.37
Deceased-donor KT	59 (41.5)	69.1 ± 20.6	68.5 ± 21.5	0.90

Continuous data are presented as means ± standard deviations; categorical data are presented as numbers (%).

AV, arteriovenous; eGFR, estimated glomerular filtration rate; KT, kidney transplantation

^a^ eGFR at the time of AV access closure

^b^ eGFR at 3 years after AV access closure

The long-term deleterious effects on right heart structure and function following AV access creation and dialysis initiation have been reported to be associated with the development of incident HF and increased risk of death despite improved control of the left ventricular pressure load through dialysis [12]. However, in the general non-transplant population, the creation of a pre-dialysis AV access has been recently suggested to delay ESKD progression [13–16]. Due to the lack of consistency across studies, there is no consensus on the causal link between AV access creation and the progression of ESKD and heart disease [3, 4].

Golper et al. [16] performed a retrospective observational analysis of a series of patients with ESKD who are not on dialysis and found that a functioning AVF may be temporarily associated with a slowing of eGFR decline and may delay the initiation of dialysis. Recently, in a nationwide cohort of 3026 US veterans with ESKD transitioning to dialysis, Sumida et al. [14] also reported that the creation of pre-dialysis AV access, such as an AVF or AVG, appears to be associated with an improvement in the rate of eGFR decline irrespective of AV access maturation and thus may delay the onset of dialysis initiation for patients with ESKD. AV access creation causes brief but repeated periods of local ischemia, thereby inducing systemic protection against tissue hypoperfusion via the pathophysiological cascades of remote ischemic preconditioning [15]. A functioning AV access also creates a low-peripheral-resistance, high-compliance venous compartment in the central arterial system [15], which may attenuate arterial stiffness and reduce both systolic and diastolic blood pressure [13, 17]. Additionally, a functioning AV access-mediated venous return enhances pulmonary flow, which may, in turn recruit larger lung areas and increase arterial oxygen content [18]. Therefore, AV access could favorably influence ESKD progression by improving oxygen delivery to the kidneys, which may attenuate the dysregulation and further loss of kidney function [15, 16, 19]. These pathophysiological mechanisms could partly explain the observations of previous studies [13–16].

However, after successful KT, controversy still exists as to whether to close or preserve a functioning AV access in KTRs with stable kidney allograft function. Hence, based on previous controversial findings [1–6, 20–22], AV access closure is not routinely recommended after successful KT but is usually performed for KTRs with specific conditions. Furthermore, there is no accepted policy for the timing of AV access closure. Nevertheless, it is important to manage KTRs with stable kidney allograft function and a functioning AV access in real-world practice. Our present findings based on a retrospective single-center analysis of 142 KTRs suggest that longitudinally observed eGFR values do not change significantly over the 3 years following the closure of a functioning AV access regardless of the type of donor graft, and the timing of AV access closure does not affect 3-year eGFR values after AV access closure.

Regarding blood pressure control and heart function, in a prospective study with 24-hour ambulatory blood pressure monitoring and left ventricular geometry assessed by echocardiography, the diastolic blood pressure was significantly increased without systolic changes at 1 month after surgical AV access closure [22]. This increase in the diastolic blood pressure was associated with a reduction of the left ventricular mass. Similarly, two prospective studies [23, 24] reported an improvement in left ventricular hypertrophy after AV access closure for KTRs with stable allograft function. Therefore, based on these observations, AV access closure has been recommended for KTRs with a stable allograft and persistent left ventricle dilatation [24]. However, the protective effect of AV access closure on long-term heart function has not been confirmed by other prospective or retrospective studies [25, 26]. A recent randomized clinical trial revealed that elective closure of AVFs in KTRs with stable kidney allograft function resulted in a significant reduction of the left ventricular mass analyzed using cardiac magnetic resonance imaging; there were no changes in the rate of eGFR or systolic and diastolic blood pressure [27]. Concerning the effect of AV access closure on kidney allograft function, a retrospective study of 311 KTRs showed that patients with a functioning AVF at 1 year after KT had significantly lower eGFR values compared with those with a spontaneously closing AVF, and AVF persistence was associated with an increased risk of kidney allograft loss in adjusted analyses [5]. Conversely, another study of 285 KTRs reported that the closure of a functioning AVF for KTRs with stable kidney allograft function significantly accelerated eGFR decline over a consecutive 12-month period after KT [3]. As a whole, the current management of a functioning AV access (surgical closure *versus* watchful preservation) for KTRs with stable kidney allograft function remains controversial and does not rely on robust evidence-based data [4, 28]. The individual risk of allograft dysfunction and a return to chronic dialysis need to be considered [4]. Recently, Laranjinha et al. analyzed the impact of functioning hemodialysis AV access on kidney allograft perfusion and found that temporary occlusion of the AV access resulted in a significant decline in kidney allograft resistive index and an increase in blood pressure [29]. Despite the small number of patients, they demonstrated, through Doppler ultrasonography, that hemodynamic changes occurred after temporary occlusion of the AV access. Large-scale prospective, multi-center clinical trials are needed.

This study had some limitations. Its retrospective design made it vulnerable to selection and information biases. A number of patients were excluded, and some clinical information (such as AV access hemodynamics, blood pressure, heart function after KT, and the reasons for AV access closure) was not available in the medical records of a substantial number of patients, which may have accounted for some of the differences in outcomes relative to other studies. This is, unfortunately, an inherent limitation of retrospective studies. There may have been indication bias in that some KTRs who were suspected of having HF were required to undergo echocardiography before and after AV access closure, which may have influenced our results. However, since there was no significant difference in the proportion of patients requiring AV access closure for the diagnosis of HF between the groups, HF *per se* is unlikely to have strongly influenced the overall results. Furthermore, the included patients were much younger than other studies, probably due to the cosmetic concern of younger patients considered for AV access closure. The effect of AV access closure might be different for older patients with a greater cardiovascular burden. This study was conducted to evaluate the pattern of the change in kidney allograft function over time after AV access closure among patients with successful KT; thus, there was no control group for comparison in this analysis. Additionally, those with early graft loss and nonfunctioning kidneys caused by postoperative immunological rejection or those who died from systemic infection were excluded from our analysis to avoid potential confounding factors; however, this may have had the effect of selection bias. The eGFR values used in this study may differ from measured GFR values. However, at our institution, we did not measure the GFR routinely; thus, we could not analyze the measured GFR values in this study. Our findings were obtained at a single center with a small sample size, which may limit the generalizability of our results; it was likely underpowered to detect the effect of certain risk factors on outcomes and to validate the association of AV access closure with changes in the eGFR. Finally, as with all observational studies, we cannot draw conclusions about causality, and our results should be considered as hypothesis-generating rather than conclusive.

In conclusion, despite the potential limitations, our current results suggest that AV access closure did not affect eGFR significantly after successful KT, and there was no significant difference in 3-year eGFR values according to the timing of AV access closure.

## Supporting information

S1 Data Data of 142 patients who underwent surgical closure of a functioning AV access after successful KT. (XLSX)
